# Astrocyte morphogenesis requires self-recognition

**DOI:** 10.21203/rs.3.rs-3932947/v1

**Published:** 2024-02-22

**Authors:** S. Zipursky, John Lee, Alina Sergeeva, Goran Ahlsen, Seetha Mannepalli, Fabiana Bahna, Kerry Goodman, Baljit Khakh, Joshua Weiner, Lawrence Shapiro, Barry Honig

**Affiliations:** UCLA/HHMI; University of California Los Angeles; Columbia University; Columbia University; Columbia University; Columbia University; Columbia University; UCLA; The University of Iowa; Columbia University; Columbia University

## Abstract

Self-recognition is a fundamental cellular process across evolution and forms the basis of neuronal self-avoidance1–4. Clustered protocadherins (Pcdh), comprising a large family of isoform-specific homophilic recognition molecules, play a pivotal role in neuronal self-avoidance required for mammalian brain development5–7. The probabilistic expression of different Pcdh isoforms confers unique identities upon neurons and forms the basis for neuronal processes to discriminate between self and non-self5,6,8. Whether this self-recognition mechanism exists in astrocytes, the other predominant cell type of the brain, remains unknown. Here, we report that a specific isoform in the Pcdhγ cluster, γC3, is highly enriched in human and murine astrocytes. Through genetic manipulation, we demonstrate that γC3 acts autonomously to regulate astrocyte morphogenesis in the mouse visual cortex. To determine if γC3 proteins act by promoting recognition between processes of the same astrocyte, we generated pairs of γC3 chimeric proteins capable of heterophilic binding to each other, but incapable of homophilic binding. Co-expressing complementary heterophilic binding isoform pairs in the same γC3 null astrocyte restored normal morphology. By contrast, chimeric γC3 proteins individually expressed in single γC3 null mutant astrocytes did not. These data establish that self-recognition is essential for astrocyte development in the mammalian brain and that, by contrast to neuronal self-recognition, a single Pcdh isoform is both necessary and sufficient for this process.

An extraordinary feature of the interactions underlying cellular morphogenesis in the brain, particularly the interactions between cellular processes, is how cells discriminate between the many different processes encountered in the developing neuropil. In the mammalian central nervous system (CNS), neurites of different neurons discriminate between their own processes and those of other neurons, in part, through the use of a large family of proteins: the clustered protocadherins (Pcdhs). These proteins regulate cell morphology and pattern neural circuits in various ways, including dendritogenesis, cell survival, and synapse formation^4,5,7–11^. In many neuronal types, Pcdhs confer upon neurons the ability to discriminate between self and non-self neurites and to promote self-avoidance, the tendency of processes of the same cells to avoid each other, thus promoting uniform coverage of receptive fields and preventing inappropriate self-synapses or autapses^12^. Pcdh genes encode on the order of 50–60 single-pass transmembrane domain proteins organized into three linked clusters: *Pcdhα, Pcdhβ*, and *Pcdhγ*^13,14^ (Fig.1a). Each isoform exhibits isoform-specific homophilic *trans* binding and is proposed to trigger a repulsive response between processes.

For Pcdh proteins to mediate self-non-self discrimination, neurons must also express different isoforms^4,5,7^. This is achieved through the probabilistic expression of multiple Pcdh isoforms in each neuron^4–6^. This endows each neuron with a unique tag, allowing neurites from the same cell to bind to each other, which, in turn, is proposed to activate repulsion, preventing them from associating with each other. Different Pcdh isoforms from any of the three clusters heterodimerize in *cis* in the plane of the plasma membrane, promoting *trans* interactions between processes expressing the same but not different combinations of isoforms^6,15,16^. As non-self processes all have different combinations of isoforms, mismatches prevent interactions between partially matching sets of Pcdh proteins on opposing membranes^6,15^. This *cis*-mediated mechanism plays a key role in regulating self-recognition specificity.

In the course of analyzing transcriptomic data from the cortex^17–19^, we were surprised to find that one Pcdh isoform, γC3, exhibited high and preferential expression in mature astrocytes in both mice and humans (Fig.1b,c). Although neurons, oligodendrocytes, and microglia in mice also express γC3, they do so at much lower levels and, in addition, express other isoforms^5,20–22^ (Fig.1b). γC3 in mouse is the predominant isoform expressed in astrocytes across 13 different brain regions^23^(Fig.1c). This isoform is also selectively expressed in human astrocytes (Fig.1c). Thus, whereas most neurons express different combinations of Pcdh isoforms in a largely probabilistic fashion^5,6^, astrocytes express γC3 in a deterministic fashion in both mouse and human. This striking difference in the mode of expression of γC3 and its conservation between mouse and human led us to assess the role of γC3 in the developing mouse visual cortex.

## γC3 is required for astrocyte morphogenesis

**Figure 2:**
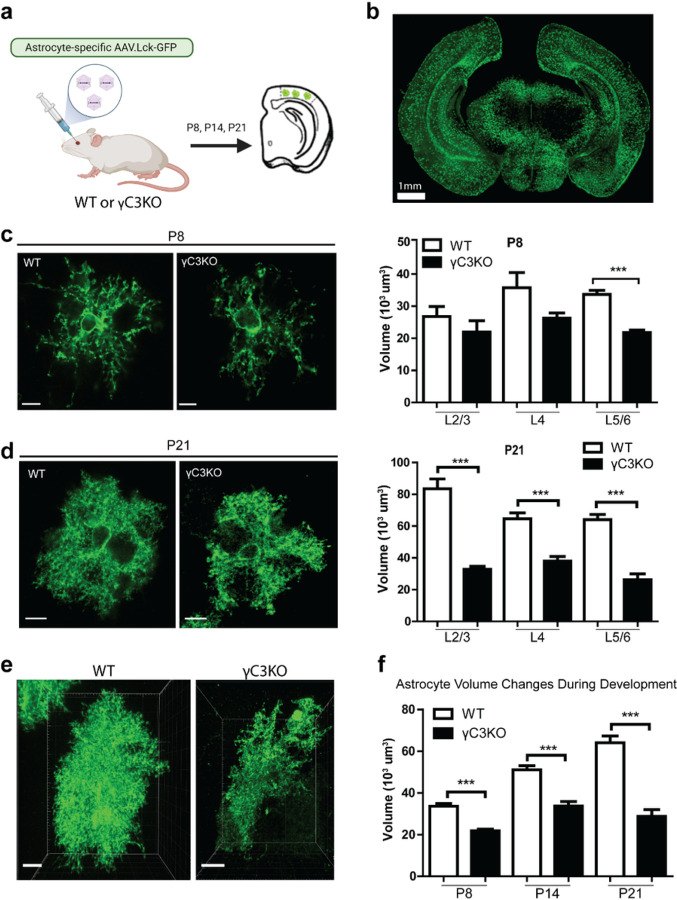
Astrocyte morphology is disrupted in γC3 KO mice. **a,** AAV vectors expressing Lck-GFP, controlled by an astrocyte-specific promoter were retro-orbitally injected into P1 neonates (see Methods). **b,** Sparse labeling of astrocytes across all cortical layers (P14 mouse cortex). **c,**Comparison of astrocyte morphology in P8 WT and γC3 KO mutants labeled with AAV expressing a myristoylated GFP (Lck-GFP) which localizes to the cytoplasmic face of the plasma membrane. Quantification of astrocyte volumes (see Methods). WT: L2/3, n=7; L4, n=8; and L5/6, n=49 astrocytes each from three mice. γC3KO mice: L2/3, n=6; L4, n=8;and L5/6, n=75 astrocytes each from three mice. **d,** Astrocytes in P21 WT and γC3KO animals. WT: L2/3, n=10; L4, n=23; and L5/6, n=35 astrocytes each from six mice. γC3KO: L2/3 n=56; L4, n=16; and L5/6 n=17 astrocytes each from six mice. **e,** Changes in astrocyte volume (see Methods) from L6 at P21. **f,** Astrocytes in L6 showed reduced volumes from P8 to P21 in γC3KO mice.P8: WT (n=48 astrocytes from three mice) and γC3KO (n=75 astrocytes from three mice); P14: WT (n=29 astrocytes from three mice) and γC3KO (n=21 astrocytes from three mice); P21: WT (n=35 astrocytes from six mice) and γC3KO (n=21 astrocytes from six mice). Error bars, s.e.m. Scale bars 10 μm. ***p<0.001.

We first sought to determine the consequence of γC3 loss in mice that lacked γC3 in all cell types^24^. γC3 knockout (KO) mice were viable and fertile and exhibited no gross signs of developmental abnormalities or neuronal cell death^24^. To explore the role of γC3 in astrocytes we sparsely labeled them through injection of PHP.eB AAVs into the retro-orbital sinus of neonatal mice at post-natal day 1 (P1) (Fig.2a). This virus, encoding a membrane targeted GFP (Lck-GFP) driven by an astrocyte-specific sequence (*GfaABC*_*1*_*D*^25,26^), drives brain-wide labeling of astrocytes^23,27^(Fig.2b). Viral delivery at P1 resulted in robust astrocyte-specific Lck-GFP expression in the visual cortex at P8, which persisted through later developmental stages (Extended Data Fig.1).

We examined the development of WT and γC3 null mutant astrocytes in fixed thick tissue preparations of V1 using confocal microscopy. We used the fast optical tissue clearing protocol (FOCM)^28^ to efficiently clear thick tissues (Extended Data Fig.2) allowing us to assess overall shape and approximate the volume of astrocytes and compared these features in different genetic backgrounds. Astrocyte processes typically undergo extensive branching between P8 and P21^29–31^ (Fig.2c–f). Abnormal morphology was seen at all stages of development (Fig.2c–f). Mutant astrocytes displayed abnormal morphology, apparent clumping of processes and reduced apparent volume (Fig.2c–f and Extended Data Fig.3a,b). These phenotypes were reminiscent of clumping of axon terminals and dendrites in different neuron types observed in deletions removing *Pcdhα* and *Pcdhγ* clusters, respectively^5,7^.

## γC3 is required cell-autonomously in astrocytes

**Figure 3:**
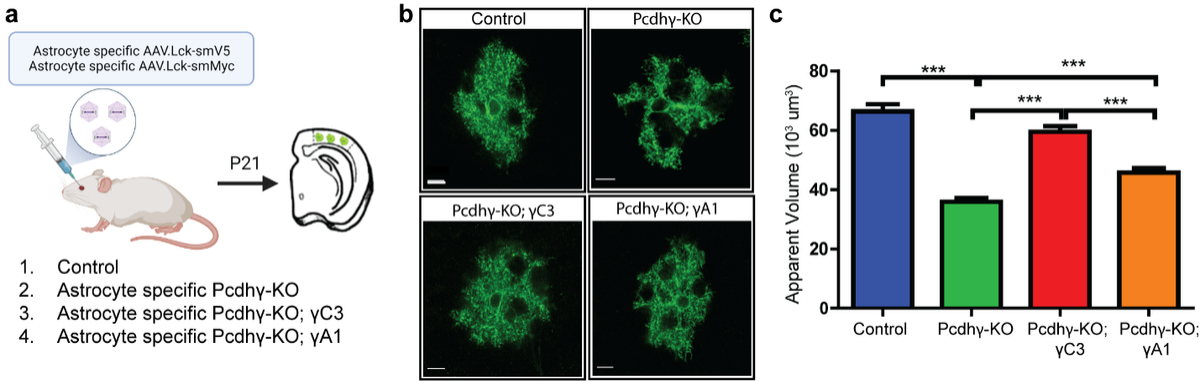
Replacement of the Pcdhγ cluster with a single isoform rescues astrocyte morphology. **a,** Strategy to determine if a single isoform alone is sufficient for normal astrocyte morphology (see text). **b,c,** Astrocytes lacking the entire Pcdhγ complex exhibit marked defects in morphology. The expression of γC3 only in astrocytes (see Methods) substantially rescues astrocyte morphological defects, while the γA1 isoform provides partial rescue. See Methods for genetic scheme. Control: n=41 astrocytes from three mice; Pcdhγ-KO, n=50 cells from three mice; Pcdhγ-KO; γC3, n=40 cells from three mice; Pcdhγ-KO; γA1, n=59 cells from five mice. Error bars, s.e.m. Scale bars, 10 μm. ***p<0.001.

We next sought to assess whether γC3 acts autonomously to regulate astrocyte morphology. To do this we selectively removed the entire Pcdhγ cluster from astrocytes using the Cre-LoxP method regulated temporally by tamoxifen and assessed morphology with and without co-expression of a γC3 cDNA in these cells (Fig.3a). Here we generated viruses expressing a spaghetti-monster fluorescent protein (smFPs) each modified with an array of small peptide epitopes to enable immunolabeling with commercially available tag-specific antibodies^32^. To enhance the labeling of fine astrocyte processes, an N-terminal membrane targeting sequence was fused to the smFP proteins enabling their targeting to the plasma membrane. The astrocyte phenotypes associated with removal of the *Pcdhγ* cluster selectively from astrocytes in early postnatal life (P1-P3) were similar to the phenotypes in γC3 KO mice (Fig.3b,c). By contrast, targeted expression of a γC3 cDNA (or to a lesser extent a PcdhγA1 (γA1) cDNA) in an astrocyte-specific Cre-dependent fashion from the ROSA26 locus in these mutant astrocytes rescued the phenotype (Fig.3b,c; for genotypes see Methods). The less efficient rescue of γA1 may reflect differences in cell surface expression levels or unique features of γC3. Importantly, these data established that γC3 is required in a cell-autonomous fashion to regulate astrocyte morphogenesis. These data are consistent with astrocyte morphogenesis relying on γC3-mediated interactions between processes of the same astrocyte or alternatively γC3-mediated interactions between astrocytes and surrounding neurons.

**Figure 4:**
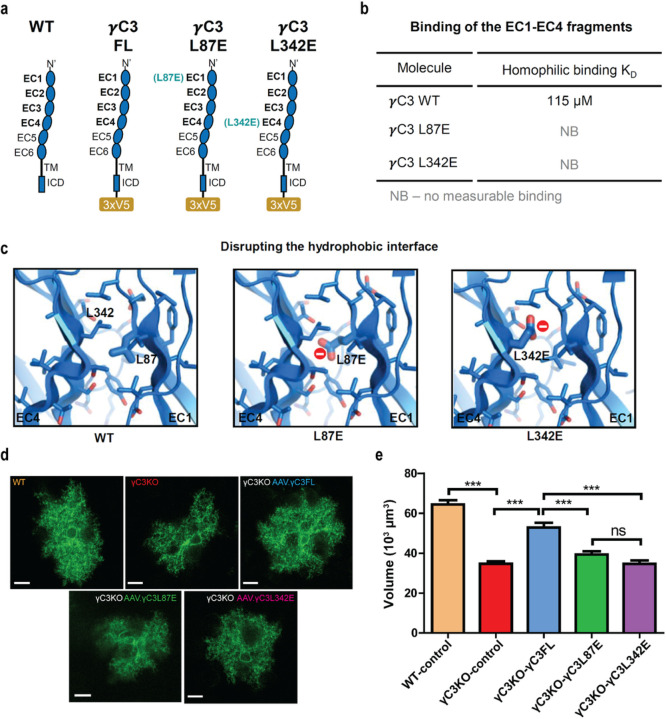
γC3 homophilic recognition specificity is required for astrocyte morphology. **a,** Schematic of proteins tested for rescue in WT and γC3 null mice. **b,** Summary of AUC experiments on the EC1-EC4 WT and mutant proteins. **c,** Structure-based design of mutations disrupting homophilic binding. Unsatisfied buried charges (red spheres) disrupt homophilic binding. **d,e,** Rescue experiments using AAV to drive different γC3 constructs under the control of the astrocyte-specific *GfaABC*_*1*_*D* promoter in WT and γC3 KO mutants. WT, n=32 cells from three animals; γC3 KO-control, n=55 cells from six animals; γC3 KO-γC3FL, n=14 cells from six animals; γC3 KO-γC3L87E, n=33 cells from six animals; and γC3 KO-γC3L342E, n=25 cells from five animals. Error bars, s.e.m. Scale bars 10 μm. ***p<0.001.

To determine whether γC3 is required in cells other than astrocytes (e.g. neurons) for normal astrocyte development, we employed a viral gene delivery strategy to selectively restore γC3 expression only in astrocytes in γC3 KO mice. We generated an AAV construct encoding a full-length γC3 cDNA fused with a C-terminal 3xV5 tag (γC3FL, Fig.4a) under the control of the astrocyte-specific *GfaABC*_*1*_*D* promoter and coinjected this into retro-orbital sinus with a virus to visualize astrocyte morphology. Astrocytes expressing γC3 (i.e. detected with the anti-V5 antibody; Extended Data Fig.4) substantially rescued the γC3 KO mutant astrocyte phenotype (Fig.4d,e). Thus, expression of γC3 molecules in astrocytes only is sufficient for astrocyte morphogenesis.

## γC3 mutants lacking homophilic binding do not rescue astrocyte morphology in γC3 mutants

If homophilic binding is required for γC3 function in astrocytes, then the genetic analysis suggests that γC3 may promote interactions between processes of the same astrocyte. It is possible, however, that γC3 recognizes a different, yet to be identified, protein on the surface of astrocytes, embedded in the extracellular matrix or on the surface of other cortical cell types that regulate astrocyte morphogenesis. If this were the case, point mutations disrupting homophilic binding would not be expected to disrupt astrocyte morphogenesis. To test this, we designed two single-point mutations in γC3 predicted to disrupt homophilic binding (Fig.4a–c). Pcdhs form *trans* dimer interfaces with their N-terminal EC1-EC4 extracellular domains binding in an anti-parallel orientation^6,15,33^. Both the EC1::EC4 and EC2::EC3 interfaces must be formed for binding to occur. To disrupt binding we designed two mutants where hydrophobic residues Leu 87 and Leu 342, which are buried within the EC1::EC4 interface, were replaced with negatively charged glutamate (Fig.4c). Both mutant proteins (γC3 EC1-EC4 L87E, and γC3 EC1-EC4 L342E) were monomers as determined by analytical ultracentrifugation (AUC) (Fig.4b). We subsequently incorporated these single point mutations into full-length γC3 proteins modified with C-terminal 3xV5 epitopes (Fig.4a). These constructs were virally introduced via retroorbital injection to transduce the cortical astrocytes of γC3 KO mice at P1 and the phenotypes were assessed at P21. Like WT γC3, mutant γC3 proteins were expressed and colocalized with membrane-tethered Lck-smMyc (Extended Data Fig.5a,b). By contrast to WT γC3, neither the γC3-L87E nor γC3-L342E mutant proteins rescued the γC3 null phenotype; the astrocytes transduced with these constructs exhibited morphology defects indistinguishable from γC3 null mutants (Fig.4d,e). These data are consistent with a requirement for γC3 to mediate interactions between processes of the same astrocytes through homophilic interactions.

## Interactions between γC3 proteins on processes of the same astrocyte are required for astrocyte morphogenesis

**Figure 5:**
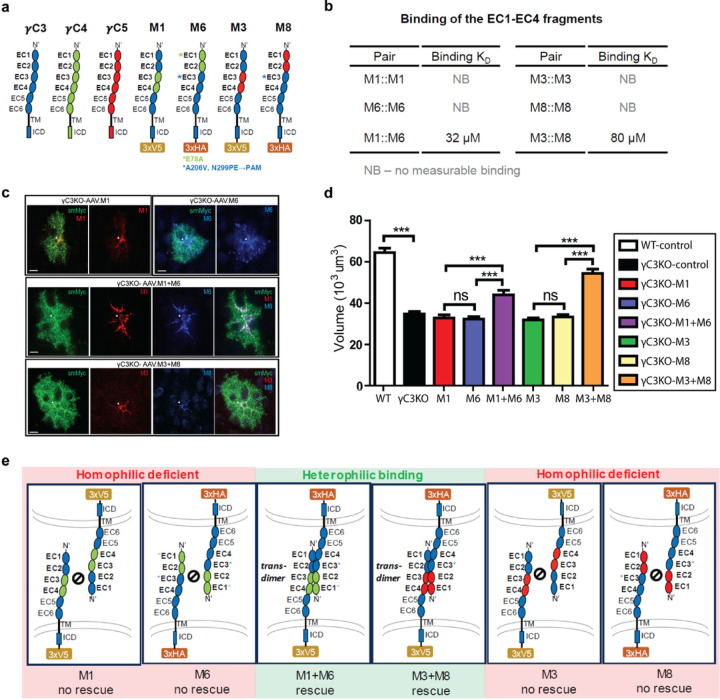
Complementary chimeras expressed in astrocytes only, rescue the morphology defect in γC3 null mutant astrocytes. **a,** Schematic showing protein constructs used in rescue experiments (see text). **b,** K_D_s for EC1-EC4 fragments of chimeras shown in A. **c,** Astrocytes transduced with virus-expressed chimeras under the control of the astrocyte-specific *GfaABC*_*1*_*D* promoter were identified by staining using antibodies to the epitope tags (arrows). Weak signal to noise was often observed with ant.i-HA antibody. Astrocyte morphology was visualized using anti-Myc staining to visualize AAV.Lck-smMyc. **d,** Quantification of astrocyte volume in different genotypes as indicated. WT, n=32 cells in three mice; γC3 KO-control, n=55 cells in six mice; M1, n=26 cells in five mice; M6, n=25 cells in five mice; M1+M6, n=19 cells in five mice; M3, n=31 cells in five mice; M8, n=20 cells in five mice; M3+M8: n=16 cells in five mice. Error bars, s.e.m. **e,** The schematic shows that complementary pairs of chimeras (M1+M6 or M3+M8) enable heterophilic binding within the same astrocytes. Scale bars, 10 μm. ***p<0.001.

To determine whether γC3 binding between processes of the same cell is required for astrocyte morphogenesis, we designed pairs of novel γC3 variants that bind to each other, but not to themselves, and tested their ability to rescue the γC3 mutant phenotype when co-expressed in the same mutant astrocyte (Fig.5). That is, we converted homophilic binding into heterophilic binding. We used a similar strategy previously to demonstrate that Dscam1 isoforms in Drosophila promote interactions between processes of the same neuron^34^.

Our approach to doing this was based, in large part, on the design of chimeras formed by combining domains of γC3 with those of either γC4 or γC5 and the introduction of additional amino acid substitutions to stabilize these interactions (Extended Data Fig.6 and 7). Swapping two domains of γC3 (blue) with two domains from γC4 (green) yielded chimeras that were monomeric in solution (Fig.5a,b for M1; and the other chimera generated in the swap shown in Extended Data Fig.7c, and Supplementary Table 1). Based on interactions between complementary domains alone we would have expected these chimeras to bind to each other (i.e. heterophilically). However, they did not (Extended Data Fig.7d).

We, thus, set out to modify the interfaces with amino acid substitutions to selectively promote heterophilic binding. This required introduction of multiple mutations into prospective heterophilic pairs guided by reiterative testing, visual inspection, and computational analysis. The detailed approach used to generate these pairs is described in Methods (Extended Data Fig.7–9 and Supplementary Table 2). Two different chimeric pairs exhibited heterophilic binding, concomitant with the loss of homophilic binding. The M1::M6 and M3::M8 pairs of the EC1-EC4 *trans* binding domain fragments had K_D_ values of 32 μM and 80 μM, respectively (Fig.5b and Supplementary Table 1). These K_D_ estimates are in the range of most cell adhesion molecules and are stronger than the K_D_ for γC3 (115 μM).

We next sought to assess whether the γC3 mutant astrocyte phenotypes were rescued by co-expression of heterophilic binding pairs. We incorporated the M1, M3, M6, and M8 EC1-EC4 chimeras into full-length γC3 protein variants. Each chimera was also tagged with a C-terminal 3xHA or 3xV5 epitope (Fig.5a) to assess expression *in vivo* using antibody staining (Fig.5c). These chimeras were expressed using the astrocyte-specific *GfaABC*_*1*_*D* promoter and incorporated into AAVs for *in vivo* expression. At P1, γC3 KO mice received injections of either a single chimera (M1, M3, M6, or M8) or a complementary pair of chimeras (M1::M6 or M3::M8 pairs) (Fig.5d). Only astrocytes positive for staining for the epitope tags were analyzed. Single chimera lacking the ability to form homodimers did not rescue the astrocyte morphology phenotype (Fig.5d). By contrast, heterophilic binding pairs (M1::M6 or M3::M8) when introduced as pairs provided substantial rescue (Fig.5d,e). Thus, interactions between γC3 proteins on the surface of processes of the same astrocyte promote normal astrocyte morphology.

## Discussion

The γC3 isoform from the *Pcdhγ* cluster is selectively expressed in both mouse and human astrocytes. We show that γC3 is necessary for astrocyte morphogenesis and through the analysis of mutant isoforms of γC3 with altered binding specificities, we provide evidence that this relies on γC3 interactions between processes of the same cell. The selective expression of γC3 in human astrocytes suggests that this function is evolutionarily conserved.

The key to establishing that γC3 mediates interactions between processes of the same astrocyte was the generation of γC3 mutants in which homophilic binding was converted to heterophilic binding. The primary challenge in generating these mutants was that different isoforms of Pcdhγ form *trans* dimers with rather distinct structures precluding simply achieving heterophilic specificity by combining different domains. Indeed, there are marked variations in surface interactions for Pcdhs (e.g. resulting in substantial deviations up to 4.5Å RMSD)^35^. Our analysis predicted that the EC2EC3 orientation in the γC3 *trans* dimer is different than that of γC4, which affects the contacts in the interface. By contrast, γC5 is expected to align closely with γC4 orientation. To accommodate these differences, we successfully created heterophilic recognition molecules by introducing targeted mutations to force the modeled chimeras to adapt *trans* dimer structures analogous to γC4.

The complexity of astrocyte morphology poses a challenge in critically assessing how γC3 contributes to astrocyte development. The loss of γC3 could lead to a collapse of astrocyte processes onto themselves or a failure of branches to segregate from each other as they extend from branch points. These phenotypes would be consistent with a repulsive function. Alternatively, interactions between astrocyte processes may be crucial to elaborate specific features of astrocyte morphology, and these interactions may be of an adhesive nature, perhaps only transiently, promoting specific interactions between processes to drive morphogenesis. A more detailed analysis of developing normal and γC3mutant astrocytes, both *in vivo* and *in vitro*, may provide ways to discriminate between these mechanisms. Regardless of the nature of the output of γC3 binding on opposed membranes, our data strongly support the conclusion that interactions between processes of the same astrocyte play a crucial role in astrocyte morphogenesis.

Although there is considerable evidence for a repulsive function of Pcdhg proteins, several studies are consistent with Pcdhg acting in an adhesive fashion. Previous studies demonstrated that the Pcdhg locus acting in astrocytes and neurons promotes the formation of both inhibitory and excitatory synapses^36^. These proteins localize in both neurons and astrocytes in close proximity to synapses^36^. It is not known, however, whether this activity is restricted to γC3 or whether this is a general property of Pcdhg proteins. In a more recent study, γC3 has been shown to promote interactions between pre- and postsynaptic neurons in the spinal cord and these are likely to be adhesive^9^. γC3 is also required in cortical pyramidal neurons to promote dendritogenesis, and the removal of γC3 from astrocytes leads to a similar phenotype in neurons^37^. This may reflect non-specific effects of changes in astrocyte morphology on dendritogenesis or, more interestingly, an adhesive function for γC3 in astrocytes directly regulating dendritogenesis in neurons. Thus, like many other cell surface axon guidance and recognition molecules, γC3 may function in an adhesive or repulsive way in a context-dependent fashion.

By contrast to the cell-type specific expression of γC3 in astrocytes, the probabilistic expression of Pcdh isoforms occurs in most neurons. As a consequence, each cell acquires a unique identity and this, in turn, allows neurites to distinguish between sister neurites (i.e. neurites of the same cell) from those of others. This typically leads to self-avoidance, allowing neurites to uniformly cover receptive fields and prevent interactions (e.g. formation of autapses) between them^5–7^. Why would astrocytes utilize γC3 selectively rather than adopt the probabilistic expression of different isoforms as neurons do? We suggest two possibilities. First, astrocytes express very high levels of γC3 transcripts, some two orders of magnitude greater than expression in neurons. This high level of expression may be necessary to support interactions between the vast number of processes and thus the massive surface area of astrocytes. Specific regulatory mechanisms may have evolved to support this function. Alternatively, as the γC3 sequences in the juxtramembrane domain are different from other Pcdhγ isoforms, these may promote interactions between astrocyte-specific proteins, which act together to pattern astrocyte morphology--either through distinct complexes on the cell surface or the activation of distinct intracellular signaling pathways. Previous studies demonstrated that a specific isoform in the Pcdha complex, PcdhαC2, is selectively expressed in serotonergic neurons and promotes axon tiling^8^. The prominent role of Pcdhs in many cell types to promote self-non-self discrimination, and the use of some isoforms in cell-type specific ways is similar to the diverse roles of different Dscam proteins in Drosophila neurodevelopment^34,38–42^.

In summary, the conservation of the selective expression of γC3 isoforms in mouse and human astrocytes, and the function we describe here for γC3 to regulate mouse astrocyte morphology argues for an evolutionarily conserved role for this isoform in regulating astrocyte development.

## Materials and Methods

### Mice

Mouse breeding and husbandry procedures were conducted in strict accordance with the guidelines and approval of UCLA’s Animal Care and Use Committee at the University of California, Los Angeles and were supervised by Josh Trachtenberg. Mice were provided with food and water ad libitum and were maintained under a 12-hour day/night cycle, with a maximum of four adult animals per cage. The PcdhγC3 knockout (γC3 KO) mice were generated and previously characterized by the laboratories of Joshua Weiner and Robert W. Burgess (The Jackson Laboratory) [21]. The Pcdhγ^fcon3/fcon3^ transgenic mice have been described previously^43^. Additionally, the ROSA26-CAG::lox-Stop-lox-PcdhγA1-mCherry and ROSA26-CAG::lox-Stop-lox-PcdhγC3-mCherry mice were originally generated by Julie Lefebvre and Joshua Sanes at Harvard University [1]. For all experiments, a minimum of three animals were analyzed per genotype.

### Tamoxifen Induction for Cre Mice

Tamoxifen (Sigma) was freshly prepared at a concentration of 20 mg/ml in corn oil and allowed to dissolve overnight at 37°C with continuous agitation. To induce gene expression, mice were injected with 5mg/kg of tamoxifen (Sigma T5648) dissolved in corn oil on postnatal day 1 or 2 for three consecutive days. This treatment aimed to eliminate Pcdhγ cluster genes and replace endogenous Pcdhγ cluster genes with either the γC3 or γA1 single isoform in astrocytes. Aldh1l1-Cre/ERT2 x Pcdhγ^fcon3/fcon3^, Aldh1l1-Cre/ERT2 x Pcdhγ^fcon3/fcon3^ x ROSA26-CAG::lox-Stop-lox-PcdhγC3-mCherry, and Aldh1l1-Cre/ERT2 x Pcdhγ^fcon3/fcon3^ x ROSA26-CAG::lox-Stop-lox-PcdhγA1-mCherry mice were used for tamoxifen injections.

### Whole-Mount Tissue Optical Clearing

To achieve optical clearing of brain tissues, we employed the FOCM reagent. The FOCM reagent was prepared as a solution containing 30% (wt/vol) urea, 20% (wt/vol) D-sorbitol, and 5% glycerol dissolved in DMSO (D8418). Mice designated for histological analysis were anesthetized with isoflurane and then transcardially perfused with ice-cold 1x PBS, followed by 4% paraformaldehyde in 1x PBS buffer. Brains were subsequently removed, postfixed in 4% paraformaldehyde overnight, and transferred to a 30% sucrose 1XPBS solution for a minimum of 48 hours at 4°C. Sections, 200 μm in thickness, were cryosectioned using a Leica cryostat microtome. These sections were washed three times for 1 hour in 1X PBS, followed by permeabilization and blocking for 4 hours in a solution containing 0.3% Triton X-100 and 10% NGS, 1% BSA, and 0.5% Triton X-100 in 1X PBS. Subsequently, slides were incubated with primary antibodies diluted in 3% NGS, 1% BSA, and 0.5% Triton X-100 in 1X PBS for 72 hours at 37°C. Primary antibodies were used at the following concentrations: Rabbit anti-V5 (Bethyl, 1:500), Chicken anti-V5 (Bethyl, 1:500), anti-c-myc rat antibody (Biorad, 1:500), Anti-GFP antibody (abcam, 1:1000). After six washes of 6 hours with 1X PBS, tissues were incubated with Alexa Fluor 488, 568, 647 secondary antibodies (Invitrogen, 1:500) or anti-RFP nanobody (FluoTag^®^-X4 anti-RFP, 1:200) for mCherry immunostaining. When necessary, tissues were treated with a nuclear counterstain, either DAPI (Invitrogen, 1:1000) or NeuroTrace Green (Invitrogen, 1:500), for 48 hours at 37°C. Finally, tissues were washed six times for 4 hours with 1X PBS and mounted on glass slides using a holder. The holder was filled with FOCM reagent, and the brain section was incubated for 15 minutes. Once the tissues became optically cleared, a glass coverslip was placed on the holder and sealed with nail polish for imaging.

### Plasmids and AAVs

To generate *GfaABC*_*1*_*D*.Lck-smV5 and Lck-smMyc plasmids, cytosolic smFP-V5 and smFP-Myc DNA sequences were amplified by PCR from the pCAG-smFP-V5 (Addgene plasmid # 59758) and pCAG-smFP-Myc plasmid (Addgene plasmid # 59757). The pCAG-smFP-V5 and pCAG-smFP-Myc plasmids were generously provided by Loren Looger. The *GfaABC*_*1*_*D*.Lck-GFP plasmid (Addgene ID #61099) was digested with restriction enzyme using XhoI and SalI. PCR amplified smFP-V5 and smFP-Myc genes were annealed into the cleavage sites of the plasmid backbone using T4 ligase to generate *GfaABC*_*1*_*D*.Lck-smV5 and Lck-smMyc plasmids, respectively. The cDNAs of the full-length γC3 fused with C-terminal 3xV5 epitopes was synthesized by Genewiz Priority Gene (Azenta Life Science). Next, the gene fragments were cut with restriction enzymes to facilitate cloning into the XhoI and XbaI digested *GfaABC*_*1*_*D*.Lck-GFP plasmid backbone to generate AAV.*GfaABC*_*1*_*D* -γC3FL construct. To generate the heterophilic binding deficient AAV chimera constructs, the EC1-EC4 domains of the WT full-length γC3 was replaced with the EC1-EC4 domains of the modified γC3 heterophilic binding chimeras (M1, M3, M6, and M8). To enable *in vivo* detection of the expressed constructs, 3xV5 tags were added to the C-terminus of the M1 and M3 constructs while 3xHA tags were fused to the C-terminus of the M6 and M8 constructs. The cDNAs for the M1, M3, M6, and M8 constructs were synthesized by Genewiz Priority Gene (Azenta Life Science). The synthesized gene fragments were restriction digested and cloned into the AAV.*GfaABC*_*1*_*D*.Lck-GFP plasmid backbone to generate AAV.*GfaABC*_*1*_*D*.M1, AAV.*GfaABC*_*1*_*D*.M3, AAV.*GfaABC*_*1*_*D*.M6, and AAV.*GfaABC*_*1*_*D*.M8 constructs.

### Generation of γC3 Single-Point Mutants

Two γC3 single-point mutants, γC3-L87E and γC3-L342E, were designed by introducing an unsatisfied negative charge at the EC1::EC4 interface of the γC3 homodimer (Fig. 4C). The biophysical characteristics of these homophilic binding deficient mutants were then assessed in the context of the γC3 EC1-EC4 construct (Table S1). QuickChange site-directed mutagenesis kit from Strategene was used to introduce point mutations on the full-length γC3 cDNA with fused C-terminal 3xV5 tags following the mutagenesis PCR protocol. The resulting AAV.GfaABC1D. γC3-L87E and AAV.GfaABC1D. γC3-L342E constructs were verified by sequencing.

### AAV Production and administration

Neonatal mice were gently removed from their holding cages and positioned on their side. Sterile saline and a cotton-tipped applicator were used to clean the injection site. A 33-gauge needle attached to the Hamilton microinjection syringe was inserted at a 45° angle into the left retro-bulbar sinus of the neonatal mice. A total injection volume of 10 μl virus diluted in 1X PBS was delivered into the retro-orbital bulbar sinus. After viral injection, mild pressure was applied at the injection site to minimize backflow of the injected viruses. AAVs were administered at an approximate viral titer of 1×10^11 vg per mouse. All injections were performed on postnatal day 1–2. Mice were sacrificed at designated time points in accordance with approved animal protocols and procedures.

To assess the effects of homophilic binding deficient mutants, separate litters of WT and γC3 KO mice each received AAV.*GfaABC*_*1*_*D*.γC3FL, AAV.*GfaABC*_*1*_*D*.γC3-L87E, or AAV.*GfaABC*_*1*_*D*.γC3-L342E. All groups were also injected with AAV.*GfaABC*_*1*_*D*.Lck-smMyc to label astrocyte morphology. In the heterophilic binding chimera experiment, the litter of γC3KO mice was equally divided to evaluate the effect of each heterophilic binding pair. To evaluate the phenotypic effect of the M1+M6 heterophilic binding chimera, the litters were equally divided to receive either AAV.*GfaABC*_*1*_*D*.M1, AAV.*GfaABC*_*1*_*D*.M6, or AAV.*GfaABC*_*1*_*D*.M1+ AAV.*GfaABC*_*1*_*D*.M6 constructs. Following a similar experimental design, other γC3 KO litters were equally divided to receive AAV.*GfaABC*_*1*_*D*.M3, AAV.*GfaABC*_*1*_*D*.M8, and AAV.*GfaABC*_*1*_*D*.M3+M8 treatments to evaluate the effect of M3 and M8 heterophilic binding pairs on astrocyte morphology. Of note, the same WT and γC3 KO control groups were shown in both Fig. 4E and Fig 5D. All AAV viruses were produced by the Janelia Viral Vector Core. Viral titers were measured using reverse transcription PCR (RT-PCR) to quantify the viral particle concentration in the final virus preparations.

### Confocal Imaging and Morphology Analysis

Confocal images were acquired by a Zeiss LSM 880 confocal microscope equipped with Zen digital imaging software. Images were acquired at several microscope magnifications including 4x, 20x, 40x with each image frame of 1024 pixels by 1024 pixels and acquired at 4x, 20x, or 40x magnifications. Z stacks images were obtained to capture the entirely of single astrocyte volume based on fluorescence signals. The acquired astrocyte z-stack confocal images were first analyzed to compute the functional surface, which was then used to extract a range of morphometric parameters for individual astrocytes. The functional surface was by creating a region of interest (ROI) that encapsulated the entirety of each individual astrocyte based on fluorescence signal. The background signal was eliminated by adjusting the threshold level. The fluorescence intensities of the GFP or smFPs used to image astrocyte morphology were equivalent across all experimental groups.For 2D morphological analysis, various morphology parameters such as Feret max, Feret min, aspect ratio, territory size, roundness, and circularity, were performed in accordance with established protocols^23,44^. This assessment was carried out using maximum intensity projections, and each morphological parameter was quantified using ImageJ software.

### Building *trans* dimer models

We used a crystal structure of γC4 (PDBID: 7JGZ)^35^ as a template to build models of *trans* dimers for γC3 wild-type (WT), γC5 WT, the single point mutants to explain their effects on binding (γC3 L87E, γC3 L342E, γC4 E78A) and the chimeras (M*i*, i =1, 2, 3, 6, 8) designed for exclusive heterophilic binding. Sequences of all modelled EC1-EC4 fragments (residues 1–421, numbering as in PDBID: 7JGZ) are juxtaposed with the template in a supplementary Fig. S10. Multiple sequence alignment shown in Fig. S10 was generated with Clustal Omega^45^ and visualized via https://espript.ibcp.fr/ESPript/ESPript/^46^.

The variants were modelled by mutating γC4 amino acids of the template structure into amino acids of the target proteins using *BuildModel* utility in FoldX^47^. This method ensures the backbone of the modeled proteins mirrors the template (γC4), gauging their propensity to form a γC4-like *trans* dimer. For instance, to build models of γC3 and γC5 wild-type *trans* dimers, we made 215 and 194 mutations to each γC4 protomer within a γC4::γC4 *trans* dimer, yielding DDG values of 27.2 and 5.9 kcal/mol, respectively. Here, DDG denotes the interaction energy of the *trans* dimer, derived from its total folding energy using *AnalyseComplex* utility in FoldX. The chimeras exhibited destabilization in a γC4-dictated *trans* dimer conformation by 16–18 kcal/mol, similar to γC3. These modelled *trans* dimers and their binding energies relative to γC4 are shown in Fig. S9A. Of note, the figure shows the precursor chimera models corresponding to merging EC1EC2 (residues 1–205) and EC3EC4 (residues of 206–421) fragments of γC3 (blue) with either γC4 (green) or γC5 (red). Only M1 and M3 match the precursor chimera in sequence. M*i* chimeras (where i=2,6,8) carry additional point mutations atop the precursor chimeras, introduced via FoldX to bolster *trans* dimerization (detailed further in ‘Design of chimeras’).

### Design of chimeras

Utilizing FoldX, we first modelled heterophilic protocadherin chimeras combining sequences of γC3 with either γC4 or γC5 (refer to ‘Building *trans* dimer models’). Subsequent mutations were introduced to correct the non-complementary surfaces. Largely positive relative FoldX energies of the initial chimera models (Fig. S9A) suggested interface issues that might hinder gC4-like heterophilic chimera binding. Particularly, the issues were likely from the γC3 fragments (as γC3::γC3 was most destabilized compared to γC4::γC4, Fig. S9A).

By examining all model structures, we identified problematic chemical interactions, especially those between polar/charged and hydrophobic residues. A comparison of these destabilizing contacts with the template interactions revealed specific issues. For instance, Fig. S9B shows a ribbon representation of a γC4 *trans* dimer (template, green) and a model of a γC3 *trans* dimer (forced to have a backbone identical to γC4, blue). The amino acids at the EC2-EC3 boundary of γC4 are mostly hydrophobic (see labelled residues shown as sticks in a diamond-shaped area, shaded in grey) whereas γC3 has polar/charged residues (N299 and E301, underlined in cyan) that are incompatible with the neighboring hydrophobic residues (L204, A118, L117). Furthermore, a hydrophobic contact in a γC4 dimer formed by two valine residues (V206) was missing in γC3 (as it has Ala in place of Val). Hence, A206, N299, and E301 residues of γC3 would likely destabilize binding in a γC4-like orientation due to polar-hydrophobic mismatches at the EC2::EC3 interface. This logic extends to chimeras containing γC3 fragments. Of note, γC5 interface is similar in chemical composition to that of γC4 featuring V206 and PAM (299–301) (Fig. S9C and Fig.S10).

To validate our observations, we employed computational mutagenesis via FoldX. We mutated residues of the precursor chimera models to make the *trans* dimer interface at the EC2-EC3 boundary more hydrophobic (see details on computational mutagenesis procedure in^48^). Predictions confirmed that A206V, N299P and E301M mutations would stabilize the interface (Table S2).

The E78A mutation enhances homophilic binding in γC4^35^. The biophysical effect of this mutation is explained in Fig.S7. The E78A mutation was introduced to M2 and M6 chimera featuring fragments of γC4. The N299P and E301M mutations were introduced to M6 and M8 chimeras together with a predicted neutral (Table S2) P300A mutation (as a triple NPE^®^PAM mutation) to minimize the number of cloning events in the chimera generation. The A206V mutation was effectively introduced by a shift in the EC2-EC3 domain boundary (i.e. we merged the domains to preserve the Val-Val contact by keeping Val206 of γC4 or γC5 and cutting out Ala206 of γC3, see Fig. S7 for the chosen domain boundary between EC1EC2 and EC3EC4 fragments in the M*i* chimeras). The NPE^®^PAM mutation differentiates M6 (with) from M2 (without) and M8 (with) from M4 (without), and it is crucial for restoring hydrophobic complementarity at the EC2-EC3 boundary in the heterophilic chimeric *trans* dimers (Fig. S9C) as M1::M6 and M3::M8 both formed heterodimers in AUC whereas M1::M2 and M2::M3 did not (Table S1).

### Sedimentation equilibrium measurements

Experiments were performed in a Beckman XL-A/I analytical ultracentrifuge (Beckman-Coulter, Palo Alto CA, USA), utilizing six-cell centerpieces with straight walls, 12 mm path length and sapphire windows. All proteins were dialyzed over-night and then diluted in TRIS 10 mM, NaCl 150 mM, CaCl2 3 mM pH 8.0, 250 mM imidazole. Samples were diluted to 1.1, 0.73 and 0.37 mg/mL in channels A, B and C, respectively. Dilution buffer were used as blank. All samples were run at four speeds, 11000, 14000, 17000 and 20000 rpm, at 25°C. The lowest speed was held for 20 h after which four measuring scans were taken with 1 h interval, the second lowest speed held for 10 h, followed by four scans as above, the third lowest and the highest speed performed identically as the second lowest speed. All measurements were done at 25°C, and detection was by interference at 675 nm. Solvent density and protein v-bar were determined using the program SednTerp. (Alliance Protein Laboratories, Corte Cancion, Thousand Oaks, CA, USA) For calculation of dimeric K_D_ and apparent molecular weight, all useful data were used in a global fit, using the program SedPhat, obtained from National Institute of Health (www.nibib.nih.gov). All measurements are summarized in Table S1.

### Protein production

cDNA for mouse Pcdh ectodomain fragments, excluding the predicted signal sequence, were cloned into a pαSHP- H mammalian expression vector (from Daniel J. Leahy, John Hopkins University), modified with the BiP signal peptide (BiP: MKLSLVAAMLLLLSAARA) and a C-terminal octahistidine tag. The signal sequence was predicted using the SignalP 4.0 server. Point mutations were introduced into the constructs using the KOD hot start polymerase (Novagen) following the standard Quikchange protocol (Stratagene). The constructs were expressed in suspension-adapted HEK293 Freestyle cells (Invitrogen) in serum-free media using polyethyleneimine as a transfectant (Polysciences). Media was supplemented with 10 mM CaCl2 4hr after transfection. Conditioned media was harvested 5 days after transfection, and the secreted proteins were purified by nickel affinity chromatography followed by size exclusion chromatography in 10 mM Tris, pH 8.0, 150 mM sodium chloride, 3 mM calcium chloride, and 250 mM imidazole, pH 8.0. The purified proteins were concentrated to > 3 mg/mL before sedimentation equilibrium AUC experiments.

### Statistical Analyses

Data were analyzed using a two-tailed Mann–Whitney test for the comparisons between two groups and one-way ANOVA analysis, followed by Tukey’s post hoc analyses, to assess for significant differences between more than two groups. All statistical analyses were performed using GraphPad Prism version 5.0c. Data are expressed as mean ± SEM. Asterisks in the figures denote the following significance levels: *p < 0.05; **p < 0.01; ***p < 0.001. In all cases, p < 0.05 was considered significant.

## Figures and Tables

**Figure 1:**
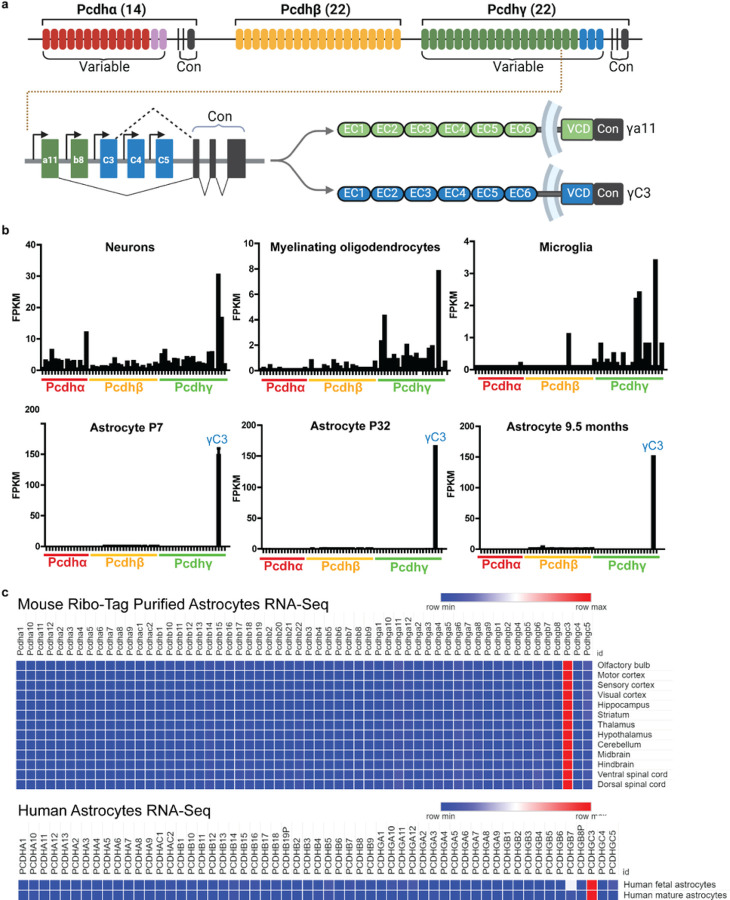
γC3 is the predominant Pcdh isoform in mouse and human astrocytes. **a,** The mouse Pcdh gene cluster contains exons encoding 58 extracellular and transmembrane domains. The α and γ proteins share common exons encoding a terminal segment of the cytoplasmic domain. Each β comprises a distinct C-terminal encoded segment. **b,** RNA-Seq transcriptional profiling of Pcdh genes in neurons, oligodendrocytes, and microglia at P7 (upper panels) and at three stages of development in astrocytes (lower panels). Note the difference in the scale on the Y-axis between the upper and lower panels. These data are from databases produced by the Barres lab (Zhang et al. 2014 and Clarke et al. 2018 available at https://brainrnaseq.org/). **c,** Expression of Pcdh genes in astrocytes from different regions of the mouse central nervous system assessed from sequences purified by the Ribo-Tag method (see Endo et al., 2022) (upper panel). Human astrocytes immunopanned from fetal (18–18.5 weeks of gestation) and adult human brains (data from Zhang et al. 2016) (lower panel). Heatmap shows the log2 FPKM values of the Pcdh cluster gene.

## Data Availability

All data are available in the main text or the supplementary materials. Further information and requests for resources and reagents should be directed to and will be fulfilled by the lead contact, S.L.Z. (lzipursky@mednet.ucla.edu).
